# Editorial: Pharmacogenomics of drug hypersensitivity reactions

**DOI:** 10.3389/fphar.2026.1913346

**Published:** 2026-06-26

**Authors:** Yaya Kassogue, Ingrid Fricke-Galindo

**Affiliations:** 1 Centre de Recherche et de Formation sur les Pathologies Moléculaires, Faculté de Médecine et d’Odonto-Stomatologie, Université des Sciences, des Techniques et des Technologies de Bamako, Bamako, Mali; 2 Pneumogenomics Laboratory, Instituto Nacional de Enfermedades Respiratorias Ismael Cosío Villegas, Mexico City, Mexico

**Keywords:** allergy, drugs, HLA, hypersensitivity, pharmacogenetics, pharmacogenomics

Drug hypersensitivity reactions (DHRs) constitute 10% of all adverse drug reactions (ADRs), and they are of significant concern due to the risk of serious morbidity and mortality (Kommu et al., 2024). The main challenge associated with in this type of ADRs is their unpredictability and the potential life-threatening nature of severe reactions (Zhang et al., 2025). Therefore, it is critically important to provide scientific information about the study of pharmacogenomic biomarkers related to these DHRs as well as the safety and economic issues involved in their implementation in clinical practice; novel diagnostic methods and genetic tests for the identification of the pharmacogenomic biomarkers, including bioinformatic studies; and relevant information for the pathophysiology of the hypersensitivity reactions.

This Research Topic addressed important unmet needs in the improvement of the drug’s safety through the compilation of research information on the mechanisms that cause these immunological responses to drugs, to improve their diagnosis, to contribute to their management strategies, and to look for those biomarkers that could predict these reactions in the future. It comprised ten manuscripts, including four reviews and six original research articles.

The first two manuscripts reviewed the pharmacogenomics of neurological drugs. First, Alvarado et al. explored the relevance of *CYP2C9* and *HLA* alleles in the pharmacokinetics, pharmacogenomics, and immunopathogenesis of the hypersensitivity reactions to antiseizure medications. The authors highlighted the available evidence supporting the usefulness of CYP2C9*3 and *HLA* alleles (i.e., HLA-A*02:01, -A*24:02, -B*13:01, -B*15:02, -B*51:01, -C*08:01) for the prediction of toxic epidermal necrolysis and Stevens-Johnson syndrome induced by phenytoin, carbamazepine, and lamotrigine, especially in Asian populations.

Then, the group of Alhaj et al. explored the pharmacogenomic biomarkers for psychiatric medications. This review presented the mechanisms underlying DHRs, considering the immunological, pharmacological, and genomic perspectives. Moreover, the authors also discussed the clinical implementation of the pharmacogenomics biomarkers in psychiatric drugs, the real-world challenges, the availability of guidelines or standardized protocols, the accessibility of genetics tests, and ethical concerns.

The two additional reviews focused on the pharmacogenomics of leukemia therapies and antibiotic-induced adverse reactions. Lameri et al. described the already validated biomarkers of *TPMT* and *NUDT15* involved in the metabolism of 6-mercaptopurine. In addition, they presented the emerging evidence relating *SLCO1B1* variants with methotrexate-induced gastrointestinal toxicity and *CEP72* with vincristine-induced neuropathy. Meanwhile, Biswas et al. reported genetic variants in *MT-RNR1* related to antibiotics such as amikacin, gentamicin, kanamycin, neomycin, tobramycin, and streptomycin; *HLA* alleles related to co-trimoxazole or flucloxacillin-induced toxicity; as well as other gene pairs like dapsone-*G6PD*, nitrofurantoin-*G6PD,* daunorubicin and doxorubicin-*RARG*, *SLC28A3*, and *UGT1A6.*

Two original articles were related to perioperative DHRs. Antonios et al. performed a retrospective study to overview the perioperative hypersensitivity reactions in Lebanon. They reported that 48.6% of the patients included in the study presented a history of perioperative reactions, with symptoms ranging from mucocutaneous manifestations to cardiopulmonary arrest. The more common drugs causing these reactions were morphine, rocuronium, and latex. In this sense, Dziadowiec et al. hypothesized that the perioperative DHRs induced by neuromuscular blocking agents could be due to genetic variants in the Mas-Related G Protein-Coupled Receptor-X2 (*MRGPRX2*); however, the authors did not find any single nucleotide variant associated with the DHRs risk, independently of the causative drug, the result of the basophil activation test, the specific IgE, or the severity of the perioperative reaction or the skin manifestations.

We also received two real-world pharmacovigilance studies. The report of Sun et al. was focused on regorafenib, a multikinase inhibitor used in oncology. They evaluated 9,442 reports of adverse events during 2012–2025 and identified a significant safety signal for regorafenib-associated hepatotoxicity, considering the elderly and male sex as risk factors. Meanwhile, the study of Li et al. analyzed severe cutaneous adverse reactions (SCARs) induced by anti-osteoporosis drugs (i.e., denosumab, alendronate, and zoledronic acid). These drugs represented 0.51% of the SCAR reports reviewed, and the authors reported that these cases occurred predominantly in females (76.25%) with a median age of 69 years. Denosumab was more commonly related to SCAR, risedronic acid with erythema multiforme, zoldronic acid with cutaneous vasculitis, and alendronic acid with Stevens-Johnson syndrome.

In addition, whole exome sequencing and HLA typing were performed by Lumkul et al. in Thai patients with confirmed beta-lactam hypersensitivity. The authors identified several candidate biomarkers for DHRs susceptibility, such as HLA-DQA1*01:04 and -DQA1*05:05. Particularly, penicillin-induced DHR was associated with HLA-C*07:02, and β-lactamase inhibitor with HLA-DQA1*01:04. Nevertheless, the further validation of the results and their replication in other ethnic populations was clearly stated in the manuscript.

Finally, Rogozina et al. centered their investigation on the diagnosis of DHRs through the lymphocyte transformation test. In a retrospective study, they compared the test with the re-exposure as the reference standard and found 100% sensitivity, 100% negative predictive value, and 78% specificity for the lymphocyte transformation test employed for the DHR diagnosis.

The manuscripts included in this Research Topic highlighted the relevance of DHRs. The efforts to improve the understanding, diagnosis, prevention, epidemiology, and management of these reactions must continue. The studies included in this Research Topic also highlighted the need to expand pharmacogenomic research beyond European and East Asian populations, particularly in underrepresented regions such as Africa and Latin America, where data on DHR and genomic susceptibility remain limited. The ultimate goal is to identify reliable biomarkers that can predict DHRs and to facilitate safer, more personalized drug therapy.
